# Oridonin attenuates the release of pro-inflammatory cytokines in lipopolysaccharide-induced RAW264.7 cells and acute lung injury

**DOI:** 10.18632/oncotarget.19249

**Published:** 2017-07-12

**Authors:** Gan Zhao, Tao Zhang, Xiaofei Ma, Kangfeng Jiang, Haichong Wu, Changwei Qiu, Mengyao Guo, Ganzhen Deng

**Affiliations:** ^1^ Department of Clinical Veterinary Medicine, College of Veterinary Medicine, Huazhong Agricultural University, Wuhan 430070, People’s Republic of China

**Keywords:** oridonin, acute lung injury, NF-κB, LPS, inflammatory cytokines

## Abstract

Acute lung injury (ALI) is a life-threatening inflammatory disease owing to the lack of specific and effective therapies. Oridonin (Ori) is an active diterpenoid isolated from *Rabdosiarubescens* (*R.rubescens*) that has been shown to possess a broadspectrum pharmacological properties including anti-inflammatory, antitumour, antioxidative and neuroregulatory effects. However, its potential protective mechanism in ALI is not well characterized. In this study, we demonstrated that Ori reduces the mortality of mice with ALI induced by a high dose of lipopolysaccharide (LPS), which suggests that Ori has a protective effect on LPS induced ALI. Next, our results confirmed that Ori improves LPS-induced localized pulmonary pathology and decreased the concentration of pro-inflammatory cytokines (IL-1β, IL-6, and TNF-α) in the serum. Nuclear factor-kappa B (NF-κB) is capable of regulating the transcription of pro-inflammatory factors. Interestingly, our results showed that Ori inhibits the expression of TLR4/MyD88 and phosphorylation of NF-κB p65 in lung tissues. To confirm this, we further validated the possible regulatory anti-inflammatory mechanisms of Ori *in vitro*. LPS-induced RAW264.7 cells, which are widely used as an inflammation model to evaluate the potential protective effect of drugs *in vitro*, were chosen for this study. Similar results were observed, that is, pre-treatment with Ori, markedly inhibited the nuclear translocation and phosphorylation of NF-κB p65 induced by LPS and subsequently decreased the release of pro-inflammatory cytokines that were increased by LPS. Overall, these results demonstrated that Ori exerts a therapeutic effect on ALI by inhibiting the release of pro-inflammatory cytokines, such as IL-1β, IL-6, and TNF-α, through the TLR4/MyD88/NF-κB axis.

## INTRODUCTION

Acute lung injury (ALI), a serious and common clinical syndrome involving acute hypoxemic respiratory failure, is a mild form of acute respiratory distress syndrome (ARDS)associated with an estimated mortality of 40–50% [1]. It can be characterized by an overwhelming inflammatory response in the lung such as recruitment of neutrophils, perivascular and interstitial oedema, elevation of microvascular permeability, disruption of epithelial integrity and impairment of gas exchange [2, 3]. In recent years, specific management protocols and supportive therapies have been developed; however, the mortality rate has not significantly improved. Therefore, exploring the molecular mechanisms of ALI and discovering novel therapeutic options are crucial.

LPS (lipopolysaccharide), the primary pathogenic factor of ALI, is an outer membrane component of gram-negative(G-)bacteria that acts as an effective initiator of local acute inflammation and is thus widely used to induce ALI in animal models for the preclinical evaluation of anti-inflammatory drug candidates [4–6]. Macrophages are widely distributed in the body and participate in immune response by providing an immediate defence against foreign agents, and their activation in response to microbial infection depends on Toll-like receptors (TLRs), which are a family of pattern recognition receptors (PRRs) involved in the regulation of both innate and adaptive immunity via the recognition of conserved microbial structures [7]. Among TLRs, TLR4has been discovered to be a sensory receptor for bacterial LPS [8]. Myeloid differentiation factor 88 (MyD88) is a key and universal downstream adapter for most TLRs that plays an important role in both innate and adaptive immune responses [9]. Following the recognition of pathogen-associated molecular patterns (PAMPs), TLRs first recruit MyD88, which induces a downstream signalling cascade and activates the NF-κB signalling eventually leading to the expression of genes encoding inflammatory factors such as tumour necrosis factor alpha (TNF-α), interleukin-6 (IL-6) and interleukin-1 beta (IL-1β) to induce a core set of inflammatory responses [10–12].

**Figure 1:**
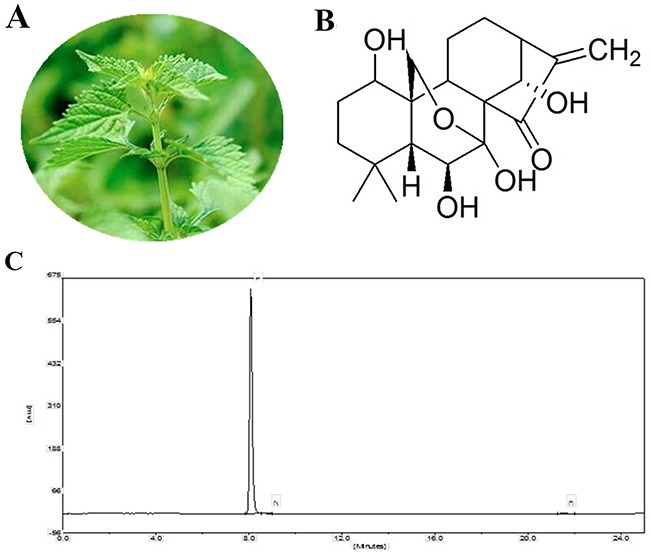
**(A)** *Rabdosiarubescens*. **(B)** Chemical structure of Ori. **(C)** HPLC chromatogram of Ori.

Traditional Chinese herbs containing a variety of natural bioactive compounds have been demonstrated to have a good effect on the treatment of various diseases [13, 14]. Ori is a natural diterpenoid isolated from the traditional Chinese herb *Rabdosiarubescens* (*R.rubescens*) (Figure 1A and 1B). Previous studies have found that Ori possessesa broad-spectrum pharmacological properties, including anti-inflammatory and neuroregulatory effects [15, 16], antitumour [17, 18] and antioxidative [16] effects. A number of studies have addressed the therapeutic potential of Ori. However, few studies have demonstrated the effects of Ori on ALI, and its potential protective mechanism is not well characterized. In this study, we investigated the effects of Ori on LPS-induced ALI *in vivo*. Additionally, we examined the effect of Ori on LPS-stimulated murine macrophages. Our findings provide experimental evidence that Ori can be used as a potential therapeutic agent in treatment for patients with lung disease.

## RESULTS

### Oridonin alleviates LPS-induced ALI

**Figure 2:**
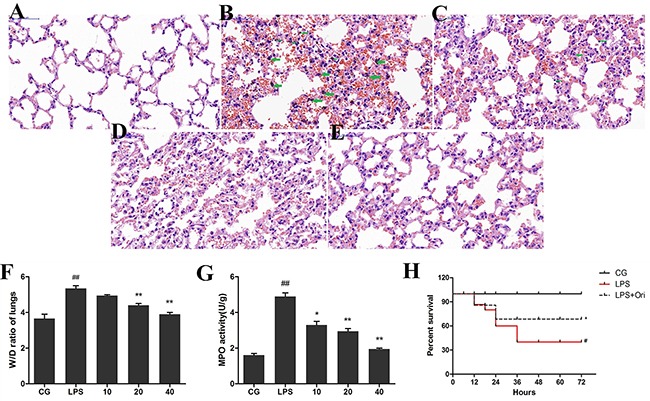
Histopathological analysisof lung tissues (HE, 200x). Lungs from each experimental group were processed for histological evaluation **(A)** Lung tissue of the control group, **(B)** the LPS group, and **(C)** to **(E)** the Ori-treated groups (10, 20, 40 μg/mL, respectively). The green arrows indicate alveolar macrophages. Effects of Ori on lung W/D ratio **(F)** and MPO activity;“g” represents tissue weight **(G)**. CG is the control group; LPS is the LPS group; and 10, 20 and 40 represent the Ori-treated groups at 10 mg/kg, 20mg/kg and 40mg/kg of Ori per animal, respectively. The values are shown as the means ± SEM (n=3). **(H)** Administration of Ori improved survival during ALI. Oridonin (Ori) was administered after establishment of LPS-induced ALI (3 mg/kg). Kaplan–Meier survival curve shows that Ori significantly improved the survival rate during 72 h compared to the LPS group. Data represent the mean ±SEM (n = 15), ^#^p< 0.05, ^##^p< 0.01 versus CG; *p< 0.05, ^**^p< 0.01 versus LPS.

Lung tissues of animals were collected, and lung injury was assessed by pathological sectioning, the wet/dry weight ratio (W/D ratio), and the MPO assay. The results of histopathology showed that the control group displayed normal tissue structures with no histopathological changes (Figure 2A). The LPS group showed severe lung injury, manifesting as inflammatory cell infiltration, alveolar wall thickening, alveolar collapse, oedema, pulmonary interstitial hyperaemia, and haemorrhage (Figure 2B). Treatment with Ori (10, 20, and 40 μg/mL) reduced the severity of lung pathology (Figure 2C to 2E, respectively). The W/D ratios showed similar results, whereas LPS dramatically increased the lung wet/dry weight ratio, confirming that pulmonary oedema occurred in the mice inoculated with LPS (Figure 2F). Ori significantly decreased the W/D ratio in a dose-dependent manner (P < 0.05 vs. LPS). To confirm the effects of Ori on LPS-induced ALI, we also measured MPO activity. The results showed that MPO activity in the LPS group was markedly increased compared with that in the control group. In contrast, MPO activity was significantly decreased in the Ori-treated groups compared with that in the LPS group (Figure 2G), which further strengthens the evidence that Ori exerts a potential protective effect against LPS-induced ALI.

### Oridonin increases the survival rate in mice with ALI

As previously described, the mice were treated with Ori (40 mg/kg, once every 8 h for a total of 3 times) or an equal volume of physiological saline after the induction of ALI (24 h after treatment with LPS at 3 mg/kg), and mortality was observed. As shown in Figure 2H, 60% of mice died within72 h in the LPS group from ALI. However, pre-treatment with Ori significantly increased the survival rate of mice within 72 h. For 40 mg/kg Ori, the survival rate increased from 40% (LPS) to 67.5% (LPS + Ori) (P < 0.05).

### Oridonin exerts negligible effect on cell viability

**Figure 3:**
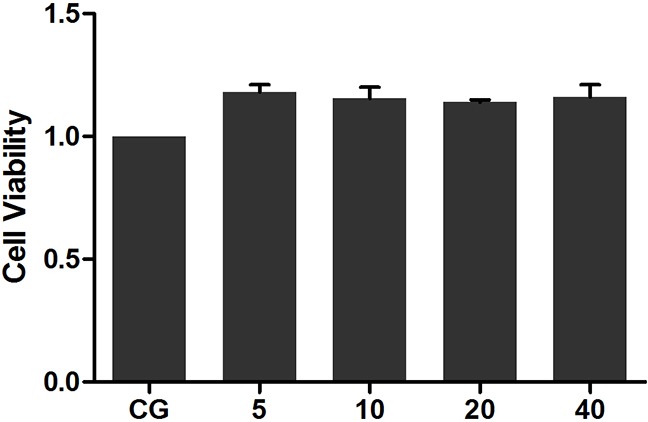
Effects of Ori on the viability of RAW 264.7 cells Cells were cultured with different concentrations of Ori (5, 10, 20, and 40 μg/mL) for 24 h. Cell viability was determined using the CCK-8 assay. CG represents the control group. The Ori-treated cell groups with 5, 10, 20 and 40 μg/mL Ori are represented as 5, 10, 20 and 40, respectively. The values presented are the means ± SEM (n=5).

The potential cytotoxicity of Ori was evaluated using the Cell Counting Kit-8 (CCK-8) assay. The results showed that cell viability was not affected by Ori (5, 10, 20 and 40 μg/mL) treatment (Figure 3, P > 0.05 vs. CG).

### Oridonin decreases the expression of pro-inflammatory cytokines

**Figure 4:**
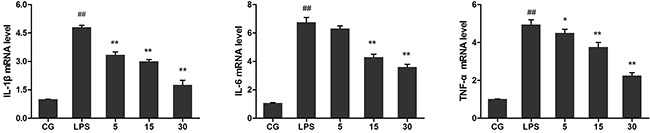
Effects of Ori on the mRNA level of TNF-α, IL-1β, and IL-6 in LPS-induced RAW 264.7 cells The cells were pre-treated with Ori (5, 15, and 30 μg/mL) for 1 h and then stimulated with LPS (1 μg/mL) for 3 h. Untreated cells served as control. qPCR was used to measure the mRNA level of TNF-α, IL-1β, andIL-6. CG represents the control group; LPS represents the LPS group; and the Ori-treated groupswith5, 15, and 30 μg/mL Ori are represented as 5, 15, and30, respectively. The values presented are the means ± SEM (n=3). ^#^p< 0.05, ^##^p< 0.01 versus CG; *p< 0.05, ^**^p< 0.01 versus LPS.

**Figure 5:**
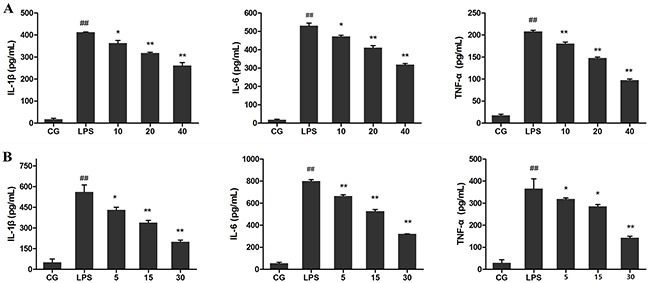
Effects of Ori on pro-inflammatory cytokines in Serum **(A)** and cell culture supernatants **(B)** CG represents the control group; LPS represents the LPS group; and the Ori-treated groups10, 20, and 40 represent 10 mg/kg, 20mg/kg and 40mg/kg Ori per animal, respectively and 5, 15, and 30 represent 5μg/mL, 15μg/mL, and 30μg/mL Ori in cells, respectively. The values presented are the means ± SEM (n=3). ^#^p< 0.05, ^##^p< 0.01 versus CG; *p< 0.05, ^**^p< 0.01 versus LPS.

Pro-inflammatory cytokines including IL-1β, IL-6, and TNF-α in the serum and cell culture supernatants were analysed using ELISA, and their mRNA levels in cells were detected using qPCR. The results showed that LPS significantly increased the mRNA level of IL-1β, IL-6, and TNF-α in RAW264.7 cells compared with those in the control group (P<0.05vs. CG). Treatment with Ori, however, reversed this trend in a dose-dependent manner (Figure 4). ELISA displayed similar results as those of qPCR, showing that stimulation with LPS led to a robust increase in TNF-α, IL-1β, and IL-6 production both in sera (Figure 5A) and cell culture supernatants (Figure 5B), which were decreased by Ori treatment in a dose-dependent manner.

### Oridonin inhibits the LPS-induced activation of TLR4/MyD88

**Figure 6:**
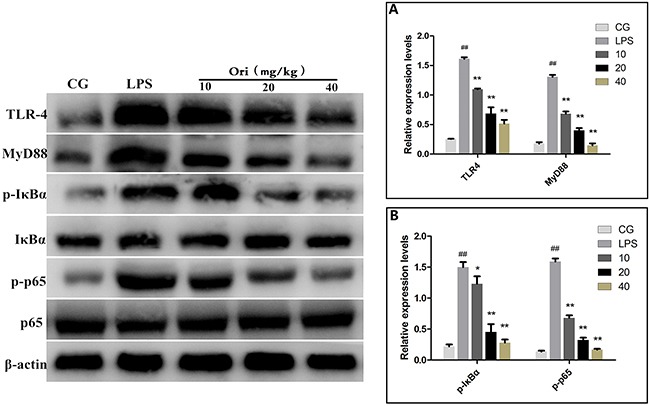
Effects of Ori onTLR4-mediated activation of the NF-κB signalling pathway in ALI Western blots were performed to detected TLR4, MyD88 **(A)** and NF-κB p65, IκBα **(B)** protein levels. β-actin or corresponding total protein (NF-κB p65 and IκBα) was used as control. CG represents the control group; LPS represents the LPS group; and the Ori-treated groups10, 20, and 40 represent 10 mg/kg, 20mg/kg and 40mg/kg of Ori per animal, respectively. The values presented are the means ± SEM (n=3). ^#^p< 0.05, ^##^p< 0.01 versus CG; *p< 0.05, ^**^p< 0.01 versus LPS.

**Figure 7:**
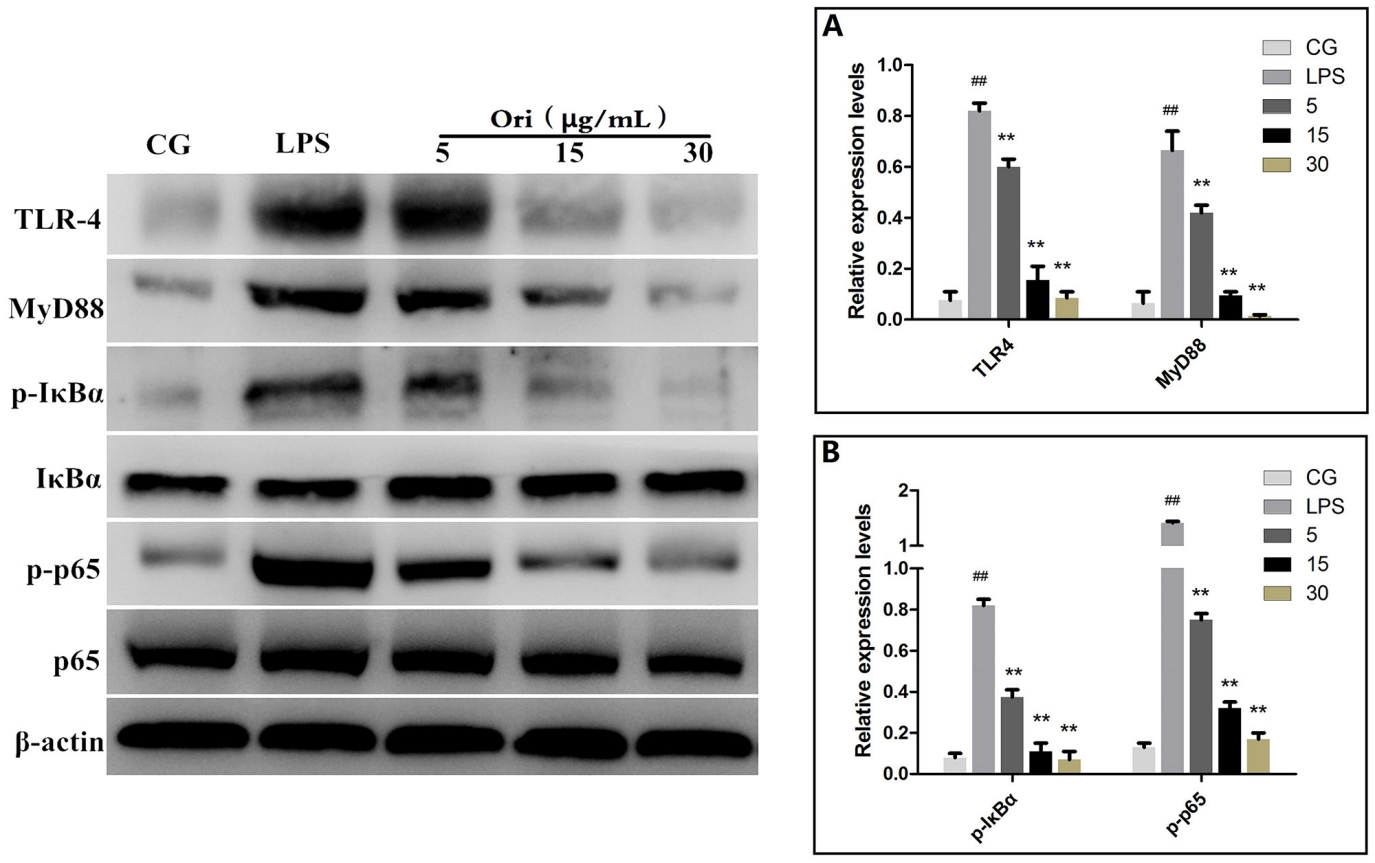
Effects of Ori on TLR4-mediated activation of the NF-κB signalling pathway in RAW 264.7 cells Western blots were performed to detected TLR4, MyD88 **(A)** and NF-κB p65, IκBα **(B)** protein levels. β-actin or corresponding total protein (NF-κB p65 and IκBα) was used as control. The Ori-treated groups 5, 15, and 30represent5, 15, and 30μg/mL Ori in cells, respectively. The values presented are the means ± SEM (n=3). ^#^p< 0.05, ^##^p< 0.01 versus CG; *p< 0.05, ^**^p< 0.01 versus LPS.

TLR4 was demonstrated to be involved in LPS-induced inflammatory processes [19]. To detect whether Ori reduced LPS-induced inflammation by inhibiting the expression level of TLR4/MyD88, western blotting was performed. The results showed that the expression of TLR4 or MyD88wasinhibited by Ori both in LPS-induced ALI (Figure 6A) and in cells (Figure 7A).

### Oridonin inhibits the LPS-induced activation of the NF-κB pathway

**Figure 8:**
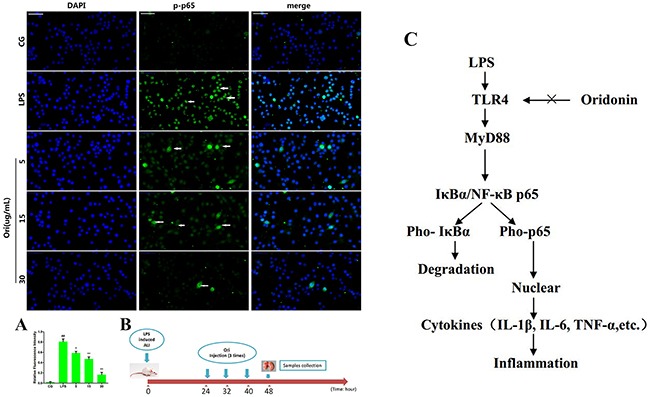
**(A)** Translocation of the p65 subunit from the cytoplasm into the nucleus was evaluated by immunofluorescence. Blue spots represent cell nuclei, and green spots represent p-p65 staining; scale bar: 50 μm. The integrated option density (IOD) of DAPI was used as an internal control. All of the data represent the mean ± SEM (n=3).^#^p< 0.05, ^##^p< 0.01 versus CG; *p< 0.05, ^**^p< 0.01 versus LPS. **(B)** Time axis of experimental animal treatment. **(C)** Schematic diagram of the signalling pathway related to the anti-inflammatory effects of Ori on LPS-induced ALI. LPS can induce NF-κB activation via TLR4-MyD88 signalling, IκBα acts as an inhibitor of NF-κB, Once the pathway is activated and IκBα is degraded, the NF-κB subunit p65 translocates from the cytoplasm to nucleus, which triggers the transcription of target genes, including TNF-α, IL-1β, and IL-6, and thus regulates inflammatory responses. However, Ori attenuates the release of pro-inflammatory cytokines by inhibiting TLR4/MyD88/NF-κB activation.

It is well known that NF-κB is a key regulator involvedin inflammatory process. Our results showed that Ori inhibits the LPS-induced activation of TLR4/MyD88. However, the activation of TLR4/MyD88 can directly affect the NF-κB pathway [19, 20]. To further confirm that Ori restricts inflammatory responses by targeting the TLR4/MyD88-mediated NF-κB signalling pathway, the nuclear translocation of NF-κB was detected using immunofluorescence assay. The immunostaining for phosphorylated NF-κB p65 (p-p65) revealed that 3 h of exposure to LPS (1 μg/mL) induced the translocation of NF-κB from the cytosol to the nucleus. However, treatment with Ori effectively blocked the nuclear translocation of NF-κB in a dose-dependent manner (Figure 8A). To further confirm the effect of Ori on the activation of NF-κB, we measured the protein expression levels of IκBα and p65 by western blotting. The results showed there was a significant increase (P < 0.05) in the phosphorylated IκBα and p65 levels in the LPS group (LPS) compared to that in the control groups(CG); however, these values were decreased by Ori treatment in a dose-dependent manner both in tissues (Figure 6B) and cells (Figure 7B). These results indicating Ori inhibits LPS-induced inflammatory responses by attenuating the release of pro-inflammatory cytokines through the inhibition ofTLR4/MyD88/NF-*κ*B activation.

## DISCUSSION

Treatment of infectious diseases and cancer has benefited from the abundance of natural products that are able to interact with many specific targets within cells, and indeed for many years, natural products have been the source of inspiration for most FDA-approved drugs [21]. Ori is an active diterpenoid isolated from *R.rubescens* that has potent anti-tumour activities in many human cancer types [22] and antioxidant properties in broiler chickens [16]. It also exhibits anti-inflammatory effects in some animal models, such as the cecal ligation and puncture (CLP)-induced septic mouse model [23]. Although Zhao et al. have reported that Ori ameliorates lung injury in CLP-induced septic mice [23], their study focused on the therapeutic effect of Ori on sepsis, and its potential protective mechanism was not well characterized. The present study was particularly aimed at studying the protective mechanism of Ori in LPS-induced ALI, which is considered a valid model to find drugs that have potential anti-inflammatory activities in various diseases.

ALI, an inflammatory disorder, is characterized by excessive inflammation. Inflammatory cascades play an essential role in the development of ALI. LPS acts as a primary infectious stimulus leading to severe inflammatory diseases, including ALI [24]. LPS-mediated murine lung injury is a disease model that shares many characteristics of sepsis-induced ALI/ARDS in humans [25]. Pulmonary oedema and excessive inflammatory cell infiltration are both characteristics of ALI [26, 27]. It has been demonstrated that activated neutrophils and alveolar macrophages cause extensive lung inflammation, destroy the basement membrane and alter the permeability of alveolar capillary membranes. The migration of neutrophils also leads to mechanical injury in the alveolar space, which further aggravates the flow of alveolar fluid [28]. MPO, known as a PMN (polymorph nuclear) marker enzyme, is used to assess the presence of multinucleated cells in the lung parenchyma andneutrophil accumulation in the alveolar space [29]. In our study, we found that LPS induced significant pulmonary oedema and aggregation and infiltration of neutrophils in mice with ALI. However, treatment with Ori significantly improved the histological changes in the lung tissues and decreased MPO activity and lung W/D ratio in mice with LPS-induced ALI. Taken together, our results demonstrated that Ori exerts a protective effect against inflammatory cell infiltration in the lungs after LPS challenge.

Macrophages plays an important role in the regulation of numerous chronic inflammatory diseases, infectious disorders and, particularly, certain autoimmune diseases by the secretion of a series of pro-inflammatory cytokines and chemokines [30, 31]. Murine macrophage RAW264.7 cells are widely used to mimic inflammation to evaluate the potential protective effect of drugs *in vitro* for conditions such as wound healing in diabetic rats [32], acute lung injury [33], and mice ear oedema [34]. Previous studies have demonstrated that macrophages release many pro-inflammatory cytokines, such as IL-1β, TNF-a and IL-6, in the early stages of inflammation induced by various pathogenic stimulants, such as LPS; excessive production of pro-inflammatory cytokines increases the extent of immune responses, which in turn results in inflammatory cascades and tissue injury [35–37]. Thus, inhibiting the release of inflammatory cytokines may be a target for anti-inflammatory drug therapies. Our results showed that Ori could significantly suppress the production of IL-1β, TNF-α and IL-6 *in vitro* and *in vivo*. Additionally, Ori did not display any cytotoxicity in RAW264.7 cells, indicating that Ori can be useful for the development of novel anti-inflammatory therapies against inflammatory diseases.

Accumulating evidence has shown that NF-κB is the key regulator of inflammatory processes. Once activated, NF-κB induces the transcription of downstream inflammatory genes, such as the pro-inflammatory molecules IL-1β, TNF-α and IL-6, [38]. LPS belongs to a prototypical class of PAMPs and is specifically recognized by TLR4 [39]. MyD88 has been shown to be canonical adaptor closely involved in inflammatory signalling pathways mediated by TLRs [40], and the transcription factor NF-κB is activated by MyD88 [41]. The activation of NF-κB and subsequent increase in the production of pro-inflammatory cytokines such as IL-1β, IL-6, and TNF-α are thought to be important for the generation of ALI [42]. In this study, our results demonstrated that Ori inhibits the expression of TLR4/MyD88 and phosphorylation of IκBα, which are increased in LPS-induced ALI mouse model. These results are consistent with those reported by a previous study [43]. This was also confirmed by *in vitro* experiments where Ori clearly inhibited the nuclear translocation and phosphorylation of NF-κB p65 in RAW264.7 cells. These results indicate that Ori exert sits potential protective effects on LPS-induced ALI by reducing the production of pro-inflammatory cytokines through theTLR4/MyD88/NF-κB signalling pathway (Figure 8C).

In conclusion, we described the therapeutic effects of Ori on LPS-induced ALI using a mouse model, as evidenced by the reduction of inflammatory cell infiltration, decrease of inflammatory cytokines and inhibition of NF-κB activation, which suggests that Ori could be a novel therapeutic option for ALI or other inflammatory diseases.

## MATERIALS AND METHODS

### Reagents

Oridonin (HPLC ≥ 98 %) was purchased from Shanghai Yuanye Biological Technology Co., Ltd. (Shanghai, China). The purity of Ori was determined by HPLC. The assay was performed on an EChrom2000 DAD Data System (Elite, Dalian, China). Chromatography was performed using a Hyper ODS-2 C18 column (5μm, 250×4.6 mm, Dikma Technology, California, USA). Elution was performed with acetonitrile/water (30:70), and the flow rate was 1.0 mL/min with DAD detection at 242 nm (Figure 1B). LPS (from Escherichia coli 055: B5) was purchased from Sigma Chemical Co. (St. Louis, MO, USA). The indicated primary antibodies and actin were obtained from Cell Signalling Technology (Beverly, MA, USA). qPCR was carried out using the SYBR Green Plus Reagent kit (Roche Applied Science, Mannheim, Germany). All of the other chemicals and reagents were of the highest commercial grade available.

### Animals and treatments

Six- to eight-week-old BALB/c mice were obtained from the Animal Experiment Center of Huazhong Agricultural University (Wuhan, China). The mice were housed at constant temperature (23°C) and relative humidity (60%) with a fixed 12 h light: 12 h dark cycle and free access to food and water. All of the experimental procedures involving animals and their care were approved by the Animal Welfare and Research Ethics Committee of Huazhong Agricultural University. Tissue collection was performed under sodium pentobarbital anaesthesia to minimize suffering.

The mice were randomly divided into five groups: control group, LPS group, and three LPS + Ori groups (10, 20, and 40 mg/kg). LPS-induced ALI was performed as our previously described [44]. Briefly, the mice were anaesthetized with an intraperitoneal injection of 100 μL of PBS containing 0.2% xylazine and 1% ketamine. Then, the mice were intranasally instilled with LPS (2 mg/kg), and the control group received equal volumes of saline. The mice were kept in a vertical position for 30 s to allow the LPS to go down the bronchoalveolar tree. After 24 h, the LPS + Ori groups were intraperitoneally injected with 10, 20, and 40 mg/kg Ori three times every 8 h (for a total of 3 times). The control group and LPS group were intraperitoneally given equal volumes of saline (Figure 8B). The mice were euthanized with CO_2_, and the serum samples and lung tissues were collected and stored at −80°C until analysis.

### Histological analysis

The lung tissues from each group were harvested and immersed in 4% paraformaldehyde, embedded in paraffin, cut into 4-μm sections, stained with haematoxylin/eosin (H&E) and then examined with a microscope (Olympus, Japan).

### Lung W/D ratio

The mice were euthanized, and the lung tissues from each group were immediately weighed and placed in an incubator at 60°C for 48 h. After complete dehydration, the dry lungs were weighed again. The ratio of wet weight and dry weight (W/D) was calculated.

### Measurement of pulmonary MPO

The right lungs from each group of mice were separated. The infiltration of neutrophils and macrophages in the lung tissue was evaluated by MPO activity as previously described [45]. MPO activity was detected with assay kits (Jiancheng Company, Nanjing, China) according to the manufacturer’s instructions, and the results were measured with a spectrophotometer at 460 nm.

### Survival curve

Based on previous experimental data, a survival curve for the different experimental groups (LPS group, control group and LPS + Ori 40 mg/kg group)was constructedat72 h after the induction of ALI. The animals that were still alive were euthanized using an excessive dose of chloral hydrate solution (0.6-0.7 mL of 5% per mouse, subcutaneous injection). After that, the mice were sacrificed by cervical dislocation.

### Cell culture and treatment

RAW264.7 cells were purchased from American Type Culture Collection (ATCC TIB-71^™^). The cells were cultured in DMEM containing 10% FBS and incubated at 37°C with 5 % CO_2_. The cells were pre-treated with Ori (5, 15, and 30 μg/mL) for 1 h and then stimulated with LPS (1 μg/mL) for 3 h. Untreated cells served as control.

### CCK-8 assay

A Cell Counting Kit-8 (CCK-8; Dojindo Laboratories) was used to assess cell viability. RAW 264.7 cells were seeded in 96-well cell culture plates at a density of 2 × 10^4^ cells/mL and then cultured with different concentrations of Ori (5, 10, 20, and 40 μg/mL) for 24 h. Subsequently, the cells were cultured with 10 μL CCK-8 in each well at 37°C for 2 h. The cell viability was measured from the absorbance (optical density) read with a microplate reader (Bio-Rad Instruments) at 450 nm and calculated using the following formula: Cell viability = (Treatment Group OD − Blank Group OD)/(Control Group OD − Blank Group OD).

### Cytokine assays

Cytokines levels (IL-1β, TNF-α and IL-6) in serum samples or cell supernatants were measured using ELISA kits according to the manufacturer’s instructions (Bio-Swamp, China).

### RNA extraction and qPCR

Total RNA was isolated using TRIzol according to the manufacturer’s recommendation (Invitrogen, USA). Then, the samples were treated with DNase I and reverse transcribed using oligo-dT primers. The total cDNA was used as starting material for real-time PCR with FastStart Universal SYBR Green Master Mix (Roche Applied Science, Germany) on a StepOne Real-time PCR System (Life Technologies Corp.). The specific primers for TNF-α, IL-1β, IL-6and GAPDH were designed based on known sequences (Table 1). The expression level of each target gene was normalized to corresponding GAPDH threshold cycle (CT) values using the 2^−ΔΔCT^ comparative method. ΔΔCt = (target gene Ct of experimental group − reference gene Ct of experimental group) − (target gene Ct of control group − reference gene Ct of control group).

**Table 1:** Oligonucleotide primers used for qPCR

Name	Accession number	Primer sequence (5′-3′)	Product size (bp)
TNF-α	NM_013693.3	Forward:CTTCTCATTCCTGCTTGTG Reverse:ACTTGGTGGTTTGCTACG	198
IL-1β	NM_008361.4	Forward:CCTGGGCTGTCCTGATGAGAG Reverse:TCCACGGGAAAGACACAGGTA	131
IL-6	NM_031168.1	Forward:GGCGGATCGGATGTTGTGAT Reverse:GGACCCCAGACAATCGGTTG	199
GAPDH	NM_001289726.1	Forward:CAATGTGTCCGTCGTGGATCT Reverse:GTCCTCAGTGTAGCCCAAGATG	124

### NF-κB p65 immunofluorescence assay

The cells were pre-treated with Ori (5, 15, and 30 μg/mL) for 1 h and then stimulated with LPS (1 μg/mL) for 3 h. Untreated cells served as control. Subsequently, cells were fixed in paraformaldehyde 4% pH 7.4 or methanol at −20°C for 3 min and then washed four times in PBS. Cells were permeabilized with 0.1% TritonX-100, exposed to the blocking solution (PBS/3% BSA) and incubated with primary antibodies against NF-κB p65 at 4°C overnight. After four washes in PBS, the cells were incubated with secondary fluorescently labelled DyLight 488 antibodies (Thermo, Rockford, USA) for 45 min at RT and washed three times in PBS. Nuclei were stained using DAPI (Roche Applied Science, Mannheim, Germany). Fluorescent images were captured with an AX70 wide-field microscope (Olympus, Japan). All morphometric measurements were observed by at least three independent individuals in a blinded manner.

### Western blot analysis

The total protein of lung tissues and cells were extracted according to the manufacturer’s recommended protocol (Vazyme, USA). The protein concentrations were determined using the BCA Protein Assay Kit (Vazyme, USA). Samples with equal amounts of protein (50 μg) were fractionated on 10% SDS polyacrylamide gels, transferred to polyvinylidene difluoride membranes, and blocked in 5% skim milk in TBST for 1.5 h at 25 ± 1°C. The membranes were then incubated at 4°C overnight with 1:1000 dilutions (v/v) of primary antibodies. After washing the membranes with TBST, incubations with 1:4000 dilutions (v/v) of secondary antibodies were conducted for 2 h at 25 ± 1°C. Protein expression was detected using the ECL Plus Western Blotting Detection System (ImageQuant LAS 4000 mini, USA). β-Actin was used as a loading control.

### Statistical analysis

The results were analysed using GraphPad Prism 5 (GraphPad InStat Software, USA). Comparisons among groups were performed with one-way ANOVA. Data were expressed as the mean ± standard error of mean (SEM). P values < 0.05 were considered statistically significant differences.

## References

[1] Phua J, Badia JR, Adhikari NK, Friedrich JO, Fowler RA, Singh JM, Scales DC, Stather DR, Li A, Jones A, Gattas DJ, Hallett D, Tomlinson G (2009). Has mortality from acute respiratory distress syndrome decreased over time? A systematic review. Am J Respir Crit Care Med.

[2] Hudson LD, Milberg JA, Anardi D, Maunder RJ (2012). Clinical risks for development of the acute respiratory distress syndrome. Am J Respir Crit Care Med.

[3] Ware LB, Matthay MA (2000). The acute respiratory distress syndrome. N Engl J Med.

[4] Brigham KL, Meyrick B (1986). Endotoxin and lung injury. Am Rev Respir Dis.

[5] Raetz CR, Whitfield C (2002). Lipopolysaccharide endotoxins. Ann Rev Biochem.

[6] Conti G, Tambalo S, Villetti G, Catinella S, Carnini C, Bassani F, Sonato N, Sbarbati A, Marzola P (2010). Evaluation of lung inflammation induced by intratracheal administration of LPS in mice: comparison between MRI and histology. MAGMA.

[7] Barton GM, Medzhitov R (2003). Toll-like receptor signaling pathways. Science.

[8] Wu H, Jiang K, Yin N, Ma X, Zhao G, Qiu C, Deng G (2017). Thymol mitigates lipopolysaccharide-induced endometritis by regulating the TLR4- and ROS-mediated NF-kappaB signaling pathways. Oncotarget.

[9] Lee KS, Scanga CA, Bachelder EM, Chen Q, Snapper CM (2007). TLR2 synergizes with both TLR4 and TLR9 for induction of the MyD88-dependent splenic cytokine and chemokine response to Streptococcus pneumoniae. Cell Immunol.

[10] Vilahur G, Badimon L (2014). Ischemia/reperfusion activates myocardial innate immune response: the key role of the toll-like receptor. Front Physiol.

[11] Baker RG, Hayden MS, Ghosh S (2011). NF-κB, inflammation, and metabolic disease. Cell Metab.

[12] Chuffa LG, Fiorucifontanelli BA, Mendes LO, Seiva FR, Martinez M, Fávaro WJ, Domeniconi RF, Pinheiro PF, Santos LD, Martinez FE (2015). Melatonin attenuates the TLR4-mediated inflammatory response through MyD88- and TRIF-dependent signaling pathways in an in vivo model of ovarian cancer. BMC Cancer.

[13] Li X, Yang X, Liu Y, Gong N, Yao W, Chen P, Qin J, Jin H, Li J, Chu R, Shan L, Zhang R, Zhang W, Wang H (2013). Japonicone A suppresses growth of Burkitt lymphoma cells through its effect on NF-kappaB. Clin Cancer Res.

[14] Koehn FE, Carter GT (2005). The evolving role of natural products in drug discovery. Nat Rev Drug Discov.

[15] Xu Y, Xue Y, Wang Y, Feng D, Lin S, Xu L (2009). Multiple-modulation effects of Oridonin on the production of proinflammatory cytokines and neurotrophic factors in LPS-activated microglia. Int Immunopharmacol.

[16] Zheng XC, Wu QJ, Song ZH, Zhang H, Zhang JF, Zhang LL, Zhang TY, Wang C, Wang T (2016). Effects of Oridonin on growth performance and oxidative stress in broilers challenged with lipopolysaccharide. Poul Sci.

[17] Huang H, Weng H, Dong B, Zhao P, Zhou H, Qu L (2017). Oridonin triggers chaperon-mediated proteasomal degradation of BCR-ABL in leukemia. Sci Rep.

[18] Shi M, Lu XJ, Zhang J, Diao H, Li G, Xu L, Wang T, Wei J, Meng W, Ma JL, Yu H, Wang YG (2016). Oridonin, a novel lysine acetyltransferases inhibitor, inhibits proliferation and induces apoptosis in gastric cancer cells through p53- and caspase-3-mediated mechanisms. Oncotarget.

[19] Fan HY, Qi D, Yu C, Zhao F, Liu T, Zhang ZK, Yang MY, Zhang LM, Chen DQ, Du Y (2016). Paeonol protects endotoxin-induced acute kidney injury: potential mechanism of inhibiting TLR4-NF-κB signal pathway. Oncotarget.

[20] Chunzhi G, Zunfeng L, Chengwei Q, Xiangmei B, Jingui Y (2016). Hyperin protects against LPS-induced acute kidney injury by inhibiting TLR4 and NLRP3 signaling pathways. Oncotarget.

[21] Mishra BB, Tiwari VK (2011). Natural products: an evolving role in future drug discovery. Eur J Med Chem.

[22] Ikezoe T, Chen SS, Tong XJ, Heber D, Taguchi H, Koeffler HP (2003). Oridonin induces growth inhibition and apoptosis of a variety of human cancer cells. Int J Oncol.

[23] Zhao YJ, Lv H, Xu PB, Zhu MM, Liu Y, Miao CH, Zhu Y (2016). Protective effects of oridonin on the sepsis in mice. Kaohsiung J Med Sci.

[24] Kovacs-Kasa A, Gorshkov BA, Kim KM, Kumar S, Black SM, Fulton DJ, Dimitropoulou C, Catravas JD, Verin AD (2016). The protective role of MLCP-mediated ERM dephosphorylation in endotoxin-induced lung injury in vitro and in vivo. Sci Rep.

[25] Marshall HE, Potts EN, Kelleher ZT, Stamler JS, Foster WM, Auten RL (2009). Protection from lipopolysaccharide-induced lung injury by augmentation of airway S-nitrosothiols. Am J Respir Crit Care Med.

[26] Schmickl CN, Biehl M, Wilson GA, Gajic O (2015). Comparison of hospital mortality and long-term survival in patients with acute lung injury/ARDS vs cardiogenic pulmonary edema. Chest.

[27] Bhargava M, Wendt CH (2012). Biomarkers in acute lung injury. Transl Res.

[28] Lakshmi SP, Reddy AT, Naik MU, Naik UP, Reddy RC (2012). Effects of JAM-A deficiency or blocking antibodies on neutrophil migration and lung injury in a murine model of ALI. Am J Physiol Lung Cell Mole Physiol.

[29] Borregaard N, Sorensen OE, Theilgaard-Monch K (2007). Neutrophil granules: a library of innate immunity proteins. Trends Immunol.

[30] Navegantes KC, de Souza Gomes R, Pereira PA, Czaikoski PG, Azevedo CH, Monteiro MC (2017). Immune modulation of some autoimmune diseases: the critical role of macrophages and neutrophils in the innate and adaptive immunity. J Transl Med.

[31] Weiss G, Schaible UE (2015). Macrophage defense mechanisms against intracellular bacteria. Immunol Rev.

[32] Zhang Q, Oh JH, Chan HP, Baek JH, Ryoo HM, Woo KM (2017). Effects of dimethyloxalylglycine-embedded poly(ε-caprolactone) fiber meshes on wound healing in diabetic rats. ACS Appl Mater Interfaces.

[33] Yang S, Yu Z, Lin W, Yuan T, Xue W, Xue Z, Wang J, Yang L, Du G (2017). The natural product bergenin ameliorates lipopolysaccharide-induced acute lung injury by inhibiting NF-kappaB activition. J Ethnopharmacol.

[34] Jung YS, Kim DH, Hwang JY, Yun NY, Lee YH, Han SB, Hwang BY, Lee MS, Jeong HS, Hong JT (2014). Anti-inflammatory effect of tricin 4’-O-(threo-β-guaiacylglyceryl) ether, a novel flavonolignan compound isolated from Njavara on in RAW264.7 cells and in ear mice edema. Toxicol Appl Pharmacol.

[35] Zhao G, Wu H, Jiang K, Chen X, Wang X, Qiu C, Guo M, Deng G (2016). The anti-inflammatory effects of interferon Tau by suppressing NF-κB/MMP9 in macrophages stimulated with staphylococcus aureus. J Interferon Cytokine Res.

[36] Wang T, Hou W, Fu Z (2017). Preventative effect of OMZ-SPT on lipopolysaccharide-induced acute lung injury and inflammation via nuclear factor-kappa B signaling in mice. Biochem Biophys Res Commun.

[37] Park JR, Lee H, Kim SI, Yang SR (2016). The tri-peptide GHK-Cu complex ameliorates lipopolysaccharide-induced acute lung injury in mice. Oncotarget.

[38] Zhao G, Jiang K, Wu H, Qiu C, Deng G, Peng X (2017). Polydatin reduces Staphylococcus aureus lipoteichoic acid-induced injury by attenuating reactive oxygen species generation and TLR2-NFκB signalling. J Cell Mole Med.

[39] Mortaz E, Adcock IM, Abedini A, Kiani A, Kazempour-Dizaji M, Movassaghi M, Garssen J (2017). The role of pattern recognition receptors in lung sarcoidosis. Eur J Pharmacol.

[40] Gorina R, Font-Nieves M, Márquez-Kisinousky L, Santalucia T, Planas AM (2011). Astrocyte TLR4 activation induces a proinflammatory environment through the interplay between MyD88-dependent NFκB signaling, MAPK, and Jak1/Stat1 pathways. Glia.

[41] Chuffa LG, Fioruci-Fontanelli BA, Mendes LO, Ferreira Seiva FR, Martinez M, Fávaro WJ, Domeniconi RF, Pinheiro PF, Delazari DS, Martinez FE (2015). Melatonin attenuates the TLR4-mediated inflammatory response through MyD88- and TRIF-dependent signaling pathways in an in vivo model of ovarian cancer. BMC Cancer.

[42] Wang J, Liu YT, Xiao L, Zhu L, Wang Q, Yan T (2014). Anti-inflammatory effects of apigenin in lipopolysaccharide-induced inflammatory in acute lung injury by suppressing COX-2 and NF-kB pathway. Inflammation.

[43] Qi J, Min Y, Qianqian G, Ciman W, Huimin W, Shanshan M, Chao L, Yeliu F, Hui J, Tong C (2015). Protective effects of polydatin on lipopolysaccharide-induced acute lung injury through TLR4-MyD88-NF-κB pathway. Int Immunopharmacol.

[44] Wu H, Zhao G, Jiang K, Chen X, Rui G, Qiu C, Guo M, Deng G (2016). IFN-tau Alleviates lipopolysaccharide-induced inflammation by suppressing NF-kappaB and MAPKs pathway activation in mice. Inflammation.

[45] Zhang Z, Zhou J, Song D, Sun Y, Liao C, Jiang X (2017). Gastrodin protects against LPS-induced acute lung injury by activating Nrf2 signaling pathway. Oncotarget.

